# Human mobility in response to COVID-19 in France, Italy and UK

**DOI:** 10.1038/s41598-021-92399-2

**Published:** 2021-06-23

**Authors:** Alessandro Galeazzi, Matteo Cinelli, Giovanni Bonaccorsi, Francesco Pierri, Ana Lucia Schmidt, Antonio Scala, Fabio Pammolli, Walter Quattrociocchi

**Affiliations:** 1 University of Brescia, Via Branze, 59, 25123 Brescia, Italy; 2 Ca’Foscari University of Venice, via Torino 155, 30172 Venezia, Italy; 3 Applico Lab-ISC CNR, Via dei Taurini 19, 00185 Rome, Italy; 4 Impact, Department of Management, Economics and Industrial Engineering, Politecnico di Milano, Milan, Italy; 5 Department of Electronics, Information and Bioengineering, Politecnico di Milano, Milan, Italy; 6 CADS, Joint Center for Analysis, Decisions and Society, Human Technopole and Politecnico di Milano, Milan, Italy

**Keywords:** Applied physics, Computer science

## Abstract

The COVID-19 pandemic is one of the defining events of our time. National Governments responded to the global crisis by implementing mobility restrictions to slow down the spread of the virus. To assess the impact of those policies on human mobility, we perform a massive comparative analysis on geolocalized data from 13 M Facebook users in France, Italy, and the UK. We find that lockdown generally affects national mobility efficiency and *smallworldness*—i.e., a substantial reduction of long-range connections in favor of local paths. The impact, however, differs among nations according to their mobility infrastructure. We find that mobility is more concentrated in France and UK and more distributed in Italy. In this paper we provide a framework to quantify the substantial impact of the mobility restrictions. We introduce a percolation model mimicking mobility network disruption and find that node persistence in the percolation process is significantly correlated with the economic and demographic characteristics of countries: areas showing higher resilience to mobility disruptions are those where Value Added per Capita and Population Density are high. Our methods and findings provide important insights to enhance preparedness for global critical events and to incorporate resilience as a relevant dimension to estimate the socio-economic consequences of mobility restriction policies.

## Introduction

The COVID-19 pandemic caused an unprecedented global health crisis with high fatality rates that stressed the national health systems and the socio-economic structures of countries^1–4^. National Governments have responded with non-pharmaceutical interventions (NPI) aimed at reducing the mobility of citizens to decrease the rate of contagion^5^. In response to the threat, the research community has exerted impressive efforts to understand on one side the epidemiological features of the outbreak^6–9^ and on the other side its economic consequences^10–12^. This unprecedented scenario calls, indeed, for a better understanding of human mobility patterns during emergencies as well as in the immediate post-disaster relief. The study of mobility habits is a foundational instance for several issues ranging from traffic forecasting, up to virus spreading, and urban planning^13–16^. However, a quantitative assessment of its statistical properties at different geographical scales remains elusive^17–26^. The availability of rich datasets on the mobility of individuals, coupled with the urgency of the current situation, has fostered the collaboration between tech giants, such as Facebook and Google, institutions, and scholars^8,27–30^. Along this path, the present work builds upon a collection of data from Facebook users and addresses the dynamics of spatial redistribution of individuals as a response to mobility restrictions applied to limit the disease outbreak. We perform a massive analysis of aggregated and de-identified data provided by Facebook through its Disease Prevention movement maps^31^ to compare the effects of lockdown measures applied in France, Italy, and the UK in response to the COVID-19 outbreak. The limitation to these three countries depends on the data available at the time of the investigation. However, the overall dataset spans over 1 month of observations and accounts for the daily movements of over 13 M people.

We model countries as networks of mobility flow and, similarly to^32^, we find that restrictions elicit geographical fragmentation through a transition toward local/short-range connections, thus causing a loss in the efficiency of mobility.

Furthermore, to quantify the substantial effect of the lockdown we provide a model to simulate the effects of movement restrictions and find that the responses to the shock observed in real mobility networks can be fairly approximated through different network dismantling strategies. Indeed, the mobility restrictions caused a general reduction of the overall efficiency in the mobility network and a geographical fragmentation with a massive reduction of long-range connections. However, different countries experience changes depending upon their initial structure of inter-connections. The three countries exhibit differentiated mobility patterns that reflect the structural diversity in their underlying infrastructure: more centralized around their capital cities in the case of France and the UK, and more clustered in the case of Italy. Such infrastructural characteristics, together with different responses to national lockdown, contributed to the emergence of varied configurations in terms of residual mobility patterns. France shows one big cluster centered in Paris and many other smaller spots that disconnect as soon as the segregation process starts. Italy exhibits four interconnected clusters, centered approximately along the high-speed rail lines in Naples, Rome, Milan, and Turin, that remain interconnected over time thus showing a high persistence and resilience. Finally, the UK has one cluster centered in London, but most of England exhibits a higher persistence with respect to France and Italy, thus suggesting the presence of more capillary infrastructures. As a further step, we try to assess the impact of mobility restrictions by coupling our modeling approach to some economic indicators. Indeed, the correlations among mobility, disease spreading, and economic variables are crucial both in emergency scenarios and in ordinary times since the different resilience of mobility networks could be both a predictor of the severity of future systemic crises and a guide to improve the economic and social impact of policies. Hence the understanding of different resilience features in national mobility networks is fundamental to craft and tailor specific release policies, and to smooth the economic impact of NPIs. To obtain a first intuition of the reasons behind the different behaviors of the three countries in our sample, we address the correlation among nodes’ persistence in the dismantling process and their economic and demographic features. We find a positive and significant relation between node persistence and, on the one side, production per capita (either measured by GDP per Capita or Value Added Per Capita) and on the other side with population density. However, the two relations are stronger for Italy and France and have a weaker effect in the UK, signaling that in this last case, as a consequence of the structural features of the capillary mobility network, also less populated and wealthy areas of Great Britain are showing high resilience to mobility disruption.

## Connectivity of national mobility networks

We represent national mobility networks of France, Italy, and the UK as weighted directed graphs, built upon movement maps provided by Facebook through their “Data for Good” program^31^ (see “Materials and methods” for further details). Notice that the analysis is limited solely to Italy, UK, and France because, at the time of the analysis, data comprising the lockdown dates were available only for these three countries.

**Figure 1:**
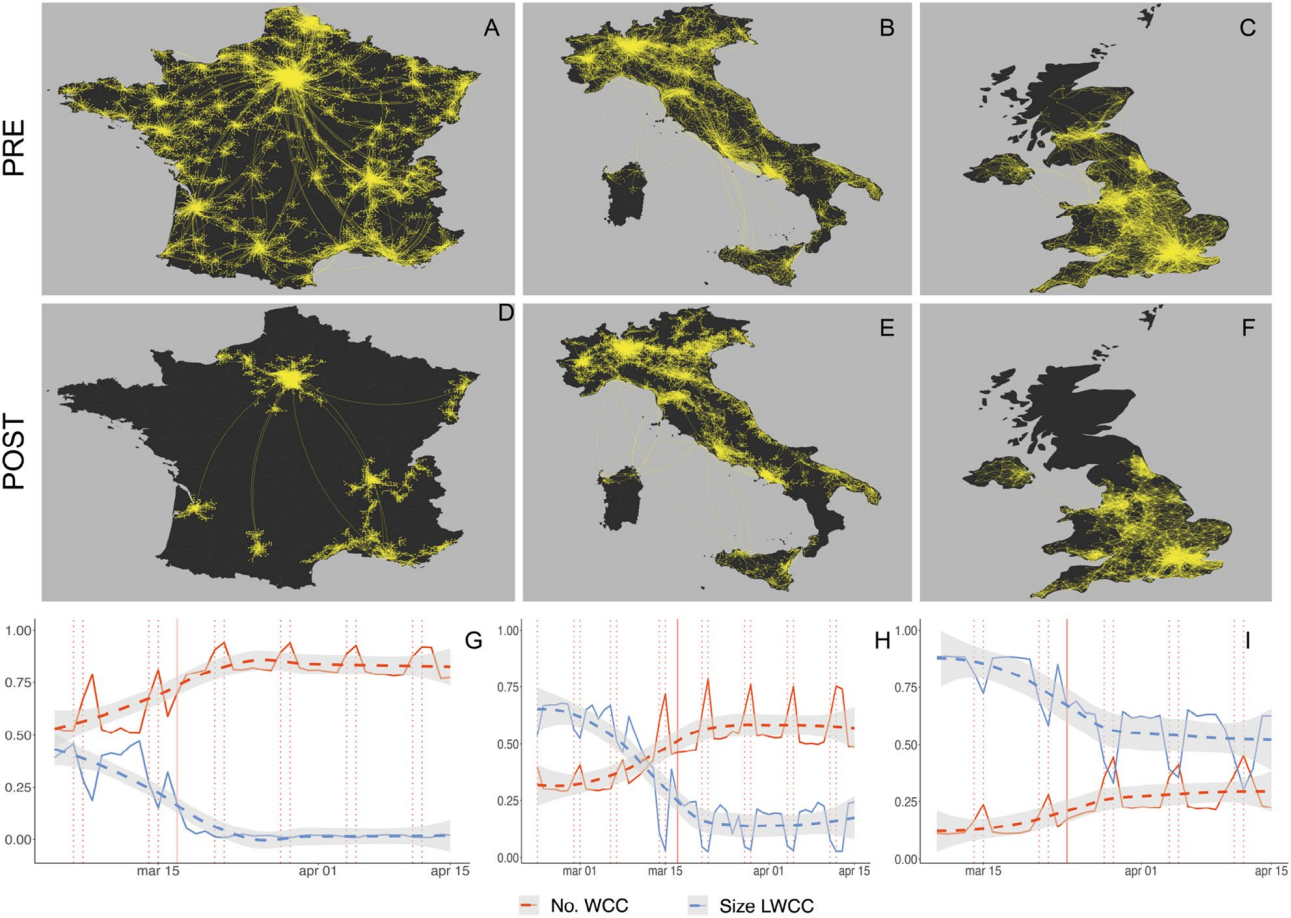
Outlook on national mobility networks for France, Italy and UK during COVID-19 pandemic. Panels (**A**–**F**) show the largest weakly connected components (LWCC) of national mobility networks built on two disjoint symmetric windows: respectively 2 weeks before (panels **A**–**C**) and 2 weeks after (**D**–**F**) the day of national lockdown. The lockdown dates are respectively March 17th for France, March 9th for Italy and March 24th for UK. Bright dots represent municipalities that belong to the LWCC. We observe the following reductions in terms of nodes that disappear from the main cluster. France: from 5495 to 1174 nodes. Italy: from 2733 to 2293 nodes. UK: from 1072 to 844 nodes. Panels (**G**–**I**) show the temporal evolution of daily connectivity for national mobility networks of municipalities, in terms of number of weakly connected components (No. WCC) and size of the largest weakly connected component (LWCC). Both quantities are normalized using the number of nodes of the corresponding network. We visualize trends by means of a LOESS regression (dashed lines with 95% confidence intervals shaded in grey) and highlight lockdown and week-end days with vertical red lines, respectively solid and dashed .

In our framework nodes correspond to municipalities and edges are weighted according to the amount of traffic between pairs of nodes. We first aggregate mobility flows in two symmetric disjoint time windows before and after the day of national lockdown (see Materials and Methods), as shown in panels (A–F) of Fig. 1. By comparing mobility networks in the period before the intervention (panels A–C of Fig. 1) and during the lockdown phase, we note a significant reduction of the overall connectivity. We find that mobility restrictions have a higher impact on the connectivity of France, whereas they yield more limited effects in the other two countries. Italy and UK, indeed, show respectively a reduction of 16% and 21% in the size of the largest weakly connected component (LWCC, i.e., the maximal subgraph of a network in which any two vertices are interconnected through an un-directed path), whereas France exhibits a reduction of almost 79%.

In panels G–I of Fig. 1, we further characterize daily connectivity patterns by computing the number of weakly connected components (No. WCC) and the size of the LWCC of mobility networks constructed on a daily basis. In all cases, we observe a decrease in the LWCC size and an increase in the number of WCCs. We also observe periodic changes in correspondence with weekends, perhaps because of the reduced necessity to commute during weekdays. In France (where the LWCC is Paris-centric), we notice a strong fragmentation of the network since the beginning: the number of WCCs is larger than the size of the LWCC, suggesting that the mobility is well distributed along with the whole country. However, Paris remains connected by long-range connections to the remaining most active areas of Bordeaux, Toulouse, Marseille, and Lyon. In the case of Italy (where the LWCC contains all the main Italian cities distributed on the high-speed rail line, i.e. Naples, Rome, Milan, and Turin), the lockdown enhances the importance of local mobility. Notice that, while in the Center and South of Italy mobility remains defined mainly at the regional level (in Fig. 1E one can distinguish Sicilia, Campania, Lazio, and Toscana), the lockdown unveils that northern Italy is more interconnected, showing a clustered mobility for the main industrial regions, i.e. Piedmont, Lombardy, Veneto, and Emilia-Romagna. The UK is clearly London-centric: the size of the LWCC remains higher than the number of WCCs, with strong local mobility patterns only in the Bristol and in Manchester-Liverpool areas. The number of WCCs and the size of the LWCC reflect the different underlying structure of the three countries: France has a hub in Paris which is star-connected via long-range links to the local city-centered areas, Italian national mobility is distributed mostly over the center-northern region, and the UK appears as an extension of London, whose network of mobility remains pervasive even after the lockdown.

## Efficiency of national mobility networks

**Figure 2:**
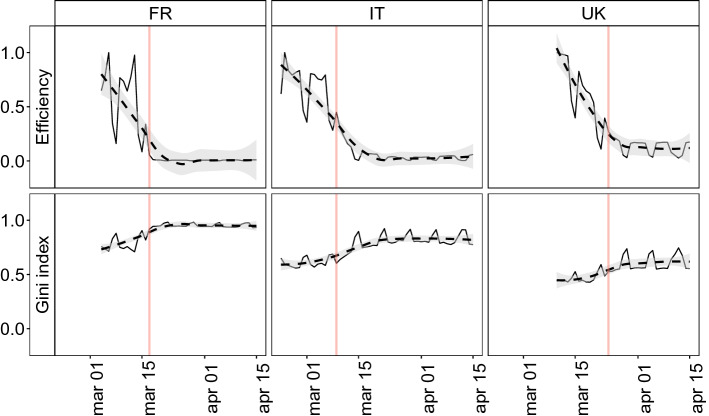
Evolution of global efficiency and of its heterogeneity. Top panels display the temporal evolution of global efficiency (normalized by its maximum value during the period of observation), for the mobility network of municipalities. We visualize trends using a LOESS regression (dashed lines with 95% confidence intervals shaded in grey) and highlight lockdown dates using a solid vertical line. Bottom panels display the temporal evolution of the Gini index of the nodal efficiency. The Gini index is used as a measure of heterogeneity and it is computed considering the nodal contributions to global network efficiency. Overall, we observe an increase of the Gini index indicating an increasing heterogeneity over time.

We further investigate the effect of lockdown focusing on the global efficiency^33^ of mobility networks, as shown in Fig. 2. The global efficiency is a measure that quantifies how optimal is the information flow in a network and it can be used as a proxy to measure its smallworldness^33^(further details are reported in “Materials and methods”). We notice a decreasing trend of the efficiency in the period before national lockdown and a steady-state in the days after the intervention (Fig. 2), which is consistent with the observed decrease in global connectivity (Fig. 1). Since the network efficiency is a measure that condensates information related to both clustering (i.e. connectedness of neighbors) and small-world effect (i.e. presence of long-range connections that act as shortcuts), the trends observed in top panels of Fig. 2 well describe the effect of an ongoing shock that cuts both long-range connections and the overall cohesiveness. Moreover, the three countries experience different amounts of decentralization as a consequence of the lockdown. To better quantify the differences, we measure the heterogeneity of nodes in terms of their contribution to global efficiency using the Gini index^34^ (see “Materials and methods”). We first observe that, in correspondence with the initial decrease of global efficiency, the Gini index displays an increasing trend that becomes steady after the lockdown date (Fig. 2). The observed trend means that the contribution of nodes to global efficiency becomes more and more heterogeneous over time until it reaches a steady-state. This result indicates a progressive disruption with very heterogeneous effects on the single nodes, suggesting that policies should be carefully tailored to avoid enhancing unequal treatments of different areas: as an example, in Italy, it has been observed that the lockdown could enhance economic disparities^29^ among municipalities. Such an effect could be even more uttered in France, which shows a higher level of heterogeneity in connectivity at municipal level with respect to Italy. Indeed, in the French context disconnected nodes are more likely to remain isolated when compared to the Italian case, which exhibits a more distributed mobility network. We observe a more nuanced response to the shock in the UK, where mobility is distributed more evenly among distinct municipalities.

## Resilience of national mobility networks

To quantify the substantial effect of the mobility restrictions applied in France, Italy, and the UK in this section, we explore the drivers behind the empirical evidence reported in previous sections. We perform an analysis based on percolation theory^35^ on the aggregated graph which corresponds to the period before the national lockdown. We assume that this setting is a good proxy for the structure of the mobility network in “business as usual” conditions (see “Materials and methods”). We implement bond percolation on the aggregated networks by iteratively deleting edges following an increasing (respectively decreasing) weight order. During the process of network dismantling, we keep track of measures related to both cohesiveness and distance, namely the LWCC size, global efficiency, and node persistence. In more detail, the node persistence measures the extent to which a node remains connected to the LWCC, that is, how much the node is resilient to percolation. Notice that several edge removal strategies can be considered to model different percolation processes and quantify different node characteristics. We tried several removal strategies based on different edge features such as weight, geographical distance, and edge betweenness. We chose edge weight removal strategy since it turns out to be the one that better approximates the real data (see “Materials and methods”).

**Figure 3:**
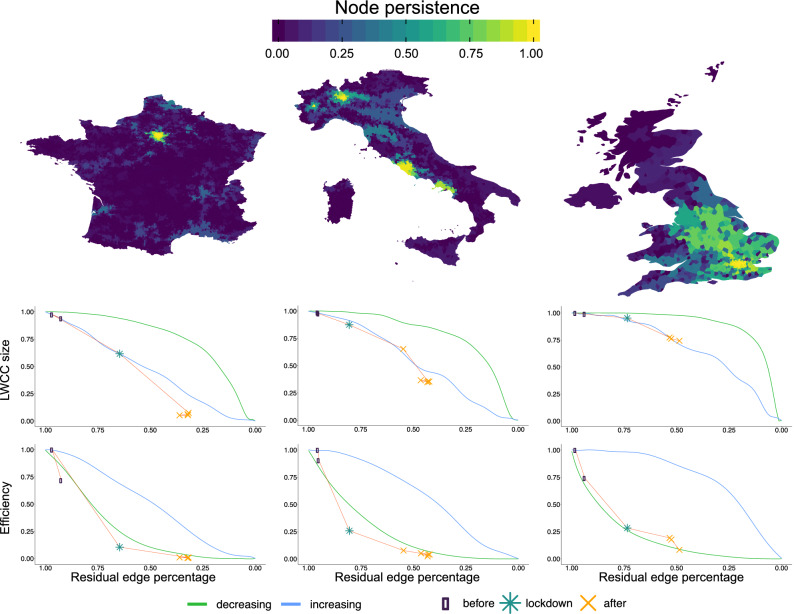
Results of the percolation process in terms of node persistence and edge removal strategies. A percolation process is performed by iterating a cutting procedure on edges according to their weights calculated in the whole period before lockdown. Top row: node persistence during the percolation process. Node persistence is defined as the number of iterations a node remains connected to the LWCC over the maximum number of iterations before the network is completely disconnected; the more persistent the node, the brighter the color. Notice that we are defining persistence upon deleting edges by their increasing strength: hence, brighter nodes are not only the last to be disconnected, but are also those embedded in stronger mobility flows. Middle row: size of the LWCC (largest weakly connected component) as a function of the number of residual edges. Green and blue curves correspond to deleting edges respectively in decreasing and increasing order of their weight. Bottom row: variation of global network efficiency obtained deleting edges by increasing (green) or decreasing (blue) weight. In both middle and bottom rows, we plot the “empirical” values of LWCC and global efficiency for the mobility networks calculated aggregating flows in the weeks before (dots), during (stars) and after (cross) the lockdown date. Notice that, while lockdown cut out the peripheries of national networks (middle panels, but see also panels (**D**–**F**) of Fig. 1), the reduced mobility severely affects also the network efficiency (bottom panels).

The top row of Fig. 3 shows the results of the percolation process in terms of node persistence, carried out by removing edges in increasing weight order for France, Italy, and the UK. To each node we assign an area on the map calculated using Voronoi tessellation^36^ and colored according to node persistence (see “Materials and methods”). The empirical evidence displayed in previous sections finds support in the percolation results. Indeed, comparing the three cases, the differences in the network structure among countries emerge. While France has one big cluster centered in Paris and many smaller modules that disconnect as soon as the process occurs, Italy exhibits four interconnected clusters, centered approximately in Naples, Rome, Milan and Turin (that roughly correspond to the high-speed rail lines), that remain interconnected and thus showing high persistence levels. Conversely, UK has one cluster centered on London, but most of England exhibits a higher persistence with respect to France and Italy, thus suggesting the presence of a more resilient network with a very capillary structure.

To compare the effects of the percolation dynamics on each of the three countries, in the middle and bottom rows of Fig. 3 we show trends for LWCC size and global efficiency throughout the process.

We first notice that the decay of the LWCC size differs depending both on the country and edge removal strategy: removing edges sorted by decreasing weight affects less the decay of the LWCC size than removing edges sorted by increasing weight, for all countries. However, we see differences in the decay of LWCC size among the three nations in the increasing case: while France exhibits an almost linear trend, UK shows a significant drop only after an important amount of connections is removed. Moreover, Italy seems to be in an intermediate situation, showing an almost linear but scattered decay. We observe that the largest connected component is quickly disrupted when edges are deleted for increasing order of weights. Following results from network dismantling theory^37^, transportation networks generally exhibit higher vulnerability to such weak link removal and are more robust to the deletion of edges with higher weight. This effect can be explained by assuming a “rich club” structure^38^, where links corresponding to higher mobility flows are concentrated around core regions. Indeed, the largest fragility of the mobility network toward the deletion of weaker edges hints at a core-periphery structure.

The bottom row of Fig. 3 displays the normalized global efficiency as a function of the residual edge. Also in this case, France has a smoother decay with respect to Italy and the UK, mainly when percolation is performed on increasing order of weights. Furthermore, when removing the same percentage of residual edges by increasing order of weights, UK yields the highest efficiency across countries. However, such a relationship changes when the opposite percolation strategy is taken into account. In this case, the UK has a slightly steeper decay of global efficiency, hinting the presence of a large number of strong connections among nodes. Again, we observe that core regions (the ones where strong links are more concentrated, i.e. the most persistent regions in the upper panels) are the most relevant. The steepest decrease in the efficiency occurs when deleting the strongest edges first. Conversely, periphery regions only mildly contribute to the network efficiency, as demonstrated by the slowest decrease upon cutting weakest edges first^37^.

To highlight the impact of the lockdown on actual mobility networks, we superimpose values in the networks observed across distinct weeks over the results of the percolation process. Each point in the curve corresponds to a certain statistic computed on the mobility network aggregated over each week (see “Materials and methods”). Mobility networks are divided into three categories based on the period taken into account: the weeks before the national lockdown, the week in which the lockdown took place, and the weeks after the lockdown.

We observe that the residual amount of edges decreases over time and it differs from country to country. France displays the lowest percentage of residual edges, while Italy and the UK tend to retain a higher percentage of their edges. Moreover, we notice how Italy and UK strongly differ in terms of the evolution of LWCC size: although they lose roughly the same fraction of edges, there is a strong difference in terms of the number of connected nodes. Despite such differences, percolation by increasing order of weights approximates adequately the decreasing trend of the LWCC size observed in all countries. The case of the normalized global efficiency is somewhat different: all countries show a similar loss proportionally to their initial efficiency. Additionally, the trend of the empirical curves is closer to that of the decreasing weight percolation strategy. The major difference observed between the middle and bottom rows of Fig. 3 is based on the fact that the considered empirical statistics follow opposite link removal strategies. This effect is due in part to the nature of the two measures; the former more related to the density and the structure of connections and the second more related to their weight. Overall, it is interesting to observe how the lockdown cannot be modeled by a removal strategy based only on edge weights. Rather, it is the result of a joint effect deriving from the removal of links based both on their structural importance and weight.

## Economic and demographic features of most resilient nodes

**Figure 4:**
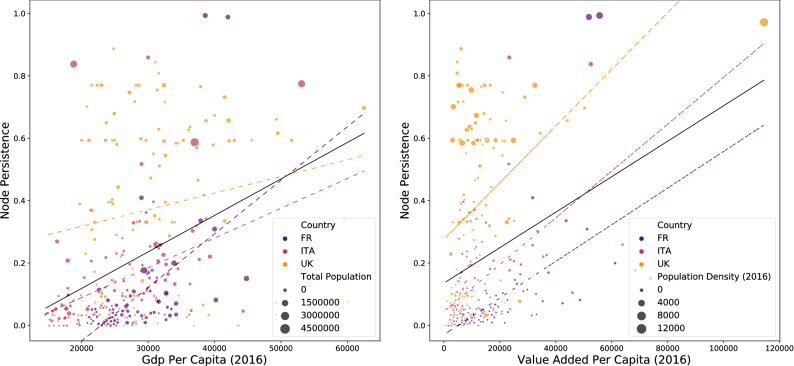
Correlation of node persistence with economic and demographic variables. Left panel: correlation between Node Persistence and GDP per Capita in 2016, with the size of nodes corresponding to Total Population in 2018. Right panel: correlation between Node Persistence and Value Added per Capita in 2016, with the size of nodes corresponding to Population Density in 2018. Overall correlation is significantly positive with respect to GDP per Capita (P–R: $P\sim 0$ $R=0.37$; S–R: $P\sim 0$ $R=0.39$; K–T: $P\sim 0$ $R=0.27$) and Valued Added per Capita (P–R: $P\sim 0$ $R=0.3$; S–R: $P\sim 0$ $R=0.4$; K–T: $P\sim 0$ $R=0.27$). Country correlations with Value Added per Capita are positive and significant for all countries, while for GDP per Capita are positive and significant only for Italy and France but not for UK (full results reported in section “Materials and methods”). Nodes have been aggregated at the province level to match economic variables. Colors of scatter points correspond to three countries in the sample. Full sample regression line reported as the continuous black line, while regressions lines for single countries are reported as colored dashed lines. Top 1% of the nodes by GDP Per Capita (left) or Value Added per Capita (right) has been removed to avoid the influence of outlier values and ease visualization (correlation results for the full sample are reported in “Materials and methods”).

As the last step in the analysis, we examine if the resilience of nodes in the network can be explained by specific economic and demographic characteristics. In the left panel of Fig. 4, we plot the relation between Gross Domestic Product (GDP) Per Capita in 2016 and Node Persistence of the municipalities in the mobility network aggregated at the province level. Colors correspond to three countries in the sample and the size of the scatter points is proportional to the total population in the province for the year 2019. To eliminate the effect of outliers we calculate correlations removing the top 1% observations by GDP (results on the full sample are reported in Table 1 of section “Materials and methods”). We find an overall positive and significant correlation among the two variables (Pearson’s R (P–R): $P\sim 0$ $R=0.37$; Spearman’s Rho (S–R): $P\sim 0$ $R=0.39$; Kendall’s Tau (K–T): $P\sim 0$ $R=0.27$) meaning that the most persistent nodes are often located in provinces where production per capita is higher, confirming the insights acquired when studying the patterns of country mobility resilience. However, by looking at the country-specific correlations (the colored dashed lines in the left panel of Fig. 4) we find that the positive relation is especially true for France and Italy while GDP per Capita in the UK has a weaker relation with Node Persistence. By looking at correlation coefficients for single countries (reported in “Materials and methods”) we note that coefficients for Italy and France are higher and significant while UK correlation is lower and significant only at a significance level between 0.1 and 0.05. This finding is explained by the different patterns of resilience in the UK which is more equally distributed across municipalities without showing the emergence of a strong core-periphery structure.

The relation between total population and persistence is instead less strong in the whole sample (P–R: $P\sim 0$ $R=0.22$; S–R: $P\sim 0$ $R=0.32$; K–T: $P\sim 0$ $R=0.21$) as also signaled by the absence of a visible pattern of the size of the scatter points. However, unlike the correlation with GDP per Capita, the weaker relation is significant both in the full sample and in the single country cases with the stronger relation found in France (P–R: $P\sim 0$ $R=0.63$; S–R: $P\sim 0$ $R=0.78$; K–T: $P\sim 0$ $R=0.60$). We can conclude that node persistence is higher in heavily populated provinces of France while in Italy and the UK the relation is less clear.

We repeat the analysis by using two different types of variables: Value Added per Capita and Population Density for the year 2016. Results are reported in the right panel of Fig. 4. As with GDP per Capita, we find a positive and significant relation between Node Persistence and Value Added per Capita (P–R: $P\sim 0$ $R=0.3$; S–R: $P\sim 0$ $R=0.4$; K–T: $P\sim 0$ $R=0.27$), this time in both the full sample and in the single country analysis and with higher correlation coefficients. We find, on average, a very strong and significant relation with Population Density in every case, as can be seen by the pattern of scatter points of greater size in the top part of the plot (P–R: $P\sim 0$ $R=0.66$; S–R: $P\sim 0$ $R=0.80$; K–T: $P\sim 0$ $R=0.61$).

We can conclude that Node Persistence is highly correlated with the wealth of provinces (either measured by Value Added Per Capita or, with a smaller effect, by GDP per Capita) and with the density and the size of the population on the territory. These effects are almost always stronger for Italy and France than for the UK. All in all, we can see how these features identify territories where urban areas and important cities create a strong modular structure with few central nodes and several peripheral zones, while in the case of the UK the territory is organized in a completely different manner. As a consequence, the economic effect of lockdown measures in these countries will certainly show a completely different spatial pattern which should be carefully analyzed.

## Conclusions

The COVID-19 pandemic is stressing the structural robustness of our societies. Most national governments have reacted to the contagion by applying mobility restrictions to contain the disease outbreak. The resulting disruption is similar to that caused by a natural disaster, but the effect is on a global scale. In this work, we analyze the effects of the lockdown in Italy, France, and the UK leveraging data from Facebook accounting for the movements of more than 13M individuals. We provide a framework to assess the effective impact of mobility restrictions and to perform a comparative analysis of the national mobility networks. We find that lockdown mainly affects national *smallworldness*—i.e., mobility restrictions yield a strong reduction of long-range connections in favor of local paths. Our analysis confirms that the national resilience to massive stress differs and depends upon the inner connectivity structure. Indeed, the three countries show very different mobility patterns that reflect the diversity of their underlying infrastructure, more concentrated in the case of France and UK, and more distributed in the case of Italy. Our analysis provides further insights into the interplay between human mobility, disease spreading and economic variables. Understanding the resilience of mobility networks can contribute to enhance the preparedness to future systemic crises and to improve our predictive capabilities on the economic and social impact of mobility restriction policies.

## Materials and methods

### Mobility data and networks

We analyzed human mobility leveraging data provided by Facebook through its “Data for Good” program^31^. The platform provides movement maps that are based on de-identified and aggregated information of Facebook users who enabled their geo-positioning.

Movements across administrative regions (i.e. municipalities in our case) are aggregated with a 8-h frequency, and describe the amount of traffic flowing between two municipalities in a given time window. Similar to data analyzed in recent research on mobility restrictions applied in China^6,7^, Facebook does not really provide the number of people moving between two locations but rather an index, constructed with a proprietary method to ensure privacy and anonymization, that highly correlates with real movements^31^.

We collected data relative to mobility in Italy, France and United Kingdom until April 15th, with different starting times depending on the availability of Facebook maps (respectively February 23th, March 10th and March 4th). The average number of daily users with their location enabled during the period of interest is 13,669,145 (France: 4,110,226; UK: 5,801,979; Italy: 3,786,940).

For the sake of our analysis we represent mobility flows using a weighted directed graph where nodes are municipalities and edges are weighted based on the amount of traffic flowing between two locations. To represent mobility networks before/during lockdown we aggregate daily traffic on windows of 12–13–14 days (respectively for France, UK and Italy) before/after the day of intervention depending on the availability of data. From these data, we build several graphs for each country. We consider a directed daily graph at municipality levels, where there can be only one edge per day from municipality A to municipality B, whose weight is the daily sum of all edges from A to B. The same procedure is applied for building aggregate networks at different time scales such as weekly graphs of pre/post lockdown graphs.

### Network efficiency

The efficiency is a global network measure that combines the information deriving from the network cohesiveness and the distance among the nodes. It measures how efficiently information is exchanged over the network^33^ and it can be defined as the average of nodal efficiencies $e_{ij}$ among couples of vertices of the network. Given a weighted network *G*(*V*, *E*) with $n=|V|$ nodes and $m=|E|$ edges, the connections of *G* are represented by the weighted adjacency matrix *W* with elements $\{w_{ij}\}$ where $w_{ij} \ge 0$ $\forall$ *i*, *j*. The global efficiency can be written by means of the following expression:

1 $$\begin{aligned} E_{glob}(G) = \frac{1}{n(n-1)}\sum _{i \ne j \in V}e_{ij} = \frac{1}{n(n-1)}\sum _{i \ne j \in G} \frac{1}{d_{ij}} \end{aligned}$$

where $d_{ij}$ is the distance between two generic nodes *i* and *j*, defined as the length of the shortest path among such nodes. The shortest path length $d_{ij}$ is the smallest sum of the weights $w_{ij}$ throughout all the possible paths in the network from *i* to *j*. When node *i* and *j* cannot be connected by any path then $d_{ij} = +\infty$ and $e_{ij} = 0$. Following the methodology of^33^, the global efficiency $E_{glob}(G)$ is normalized in order to assume maximum value $E(G)=1$ in the case of perfect efficiency. In such a setting the nodal efficiency, i.e. the contribution of each node to the global efficiency, can be simply written as:

2 $$\begin{aligned} e_{i} = \frac{1}{n-1}\sum _{j \ne i}\frac{1}{d_{ij}} \,. \end{aligned}$$

Beside the geographical distance between two nodes of the graphs, proximity can also be defined considering that two locations are closer if many movements happen between them. To compute network efficiency in our case we use the reciprocal of weights on links to obtain the shortest path distance among couples of nodes.

### Gini index

The Gini index is a classic example of a synthetic indicator used for measuring inequality of social and economic conditions. The Gini index can be defined starting from the Gini absolute mean difference $\Delta$^39^ of a generic vector *y* with *n* elements, that can be written as:

3 $$\begin{aligned} \Delta = \frac{1}{n^2}\sum _{i=1}^n \sum _{j=1}^n |y_{i} - y_{j}| \end{aligned}$$

The relative mean difference is consequently defined as $\Delta /\mu _y$ where $\mu _y= n^{-1}\sum _{i=1}^n y_i$. Thus, the relative mean difference equals the absolute mean difference divided by the mean of the vector *y*. The Gini index *g* is one-half of the Gini relative mean difference.

4 $$\begin{aligned} g = \frac{\Delta }{2 \mu _y} \end{aligned}$$

Values of $g \sim 1$ signal that the considered vector displays high inequality in the distribution of its entries, while values of $g \sim 0$ signal a tendency towards equality.

### Percolation process and node persistence

To measure the extent to which a node resists to percolation process, we define the following quantity as node persistence. Consider a graph *G* with nodes *V* = $\{v_1,\dots ,v_k\}$ and edges *E *= $\{e_1,\dots ,e_h\}$. Let $S:E \mapsto {\mathbb {R}}^+$ be a function that assign a finite positive real number $w_l$, $l \in 1\dots h$ to each edges $e_1,\dots ,e_h$ of the network. For instance, *S* can be the function that assigns each edge its weight or its position in the edge ranking for a certain metric, e.g edge betweenness. More simply, *S* can be seen as a function that assigns a removal order to the edges. Consider the finite set $W=[w_1,\dots ,w_{n+1}]$ of the distinct values taken by $w_l$, with $w_1<w_2<\dots <w_{n+1}$. Suppose that we run a percolation process that consists of sequentially deleting edges according to the values assigned by *S* to each edge in increasing order, that is, in the first iteration we delete all the edges with $w_l$ less or equal than $w_1$, in the second iteration we delete all the edges with weights less or equal than $w_2$ and so on until the last iteration, $n+1$, where we delete all the edges in the network.

Notice that there is no loss of generality in considering only increasing order, since the *S* function can be arbitrarily defined.

Thus, at iteration *i*, we delete all the edges with $w_l$ less or equal than $w_i$. Consider now a node $v_j$ $\in$ *V*. At each step of the process, $v_j$ may or may not be part of the LWCC of the network *G*. Clearly, if $v_j$ does not belong to the LWCC of *G* at step *i*, it will not be in the LWCC of *G* at step $i+1,i+2,\dots ,n+1$. Defining $M_{v_j}$ as the maximum number of iterations *i* such that $v_j$ belongs to the LWCC of *G*, the persistence of node $v_j$ respect to the ordering induced by function *S* is defined as:

5 $$\begin{aligned} \rho =\frac{M_{v_j}}{n} \end{aligned}$$

That is, if the whole process takes $n+1$ iterations to disconnect the whole network, the persistence of a node $v_j$ respect to the function *S* is defined as the maximum number of iterations for which $v_j$ is connected to the LWCC over the maximum number of iteration for which there are connected nodes in the network.

Notice that several instances of the function S and several edge removal strategies can be considered to model different percolation processes. We tried several removal strategies based on different edge features such as weight, geographical distance, and edge betweenness. Since our goal is to mimic the disruption of the countries’ mobility networks, we focus on the one that better approximates the networks’ connectedness over time, which turned out to be increasing edge weight (Supplementary Information 1).

### Correlation of economic and demographic indicators with nodes persistence

**Table 1:** Correlation among node persistence and economic and demographic indicators calculated for all countries together and separately on each country subset.

	Country	All observations			Obs	Without top 1%			Obs
		Pearson	Spearman	Kendall		Pearson	Spearman	Kendall	
Gdp Per Capita (2016)	All	0.3046	0.4154	0.2894	328	0.3732	0.3962	0.2745	324
	ITA	0.4305	0.5374	0.3866	110	0.4305	0.5374	0.3866	110
	FR	0.7751	0.5409	0.3836	93	0.5655	0.5101	0.3574	91
	UK	0.2294	0.2148	0.1529	125	0.1795	0.1964	0.1406	123
		*0.0101*	*0.0162*	*0.0122*		*0.0469*	*0.0295*	*0.0224*	
Total population (2019)	All	0.2766	0.3344	0.2273	329	0.2234	0.3179	0.2152	325
	ITA	0.6543	0.4939	0.3581	110	0.3706	0.4507	0.3235	107
	FR	0.6035	0.7921	0.605	94	0.6358	0.7862	0.6001	93
	UK	0.4544	0.5095	0.3603	125	0.4544	0.5095	0.3603	125
Value added per capita (2016)	All	0.398	0.4215	0.2917	328	0.3059	0.402	0.2762	324
	ITA	0.639	0.5987	0.4369	110	0.5433	0.5764	0.4171	108
	FR	0.794	0.7801	0.5954	93	0.6012	0.7655	0.5783	91
	UK	0.4214	0.5203	0.3744	125	0.4214	0.5203	0.3744	125
Population density (2016)	All	0.5966	0.813	0.622	329	0.6594	0.806	0.6128	325
	ITA	0.8303	0.6612	0.4863	110	0.8303	0.6612	0.4863	110
	FR	0.8292	0.8542	0.6794	94	0.9265	0.8394	0.6588	91
	UK	0.5311	0.6985	0.5079	125	0.517	0.6911	0.4998	124

Calculation performed on the full sample (columns on the left) and on the sample without the top percentile to avoid the influence of outliers. First row reports correlation coefficients, second row reports in italic p values only for values greater than 0.001.

## Supplementary Information

Supplementary Information 1.

## Data Availability

For what concerns Facebook human mobility, all data are provided under an academic license agreement with Facebook through its “Data for Good” program (available at https://dataforgood.fb.com/tools/disease-prevention-maps/). Facebook releases data upon request to nonprofit organization and academics. Economic data have been obtained from Eurostat and are available at https://ec.europa.eu/eurostat/data/database.
