# Supplementary material for: Human mobility in response to COVID-19 in France, Italy and UK
Source: Sci Rep. 2021 Jun 23;11:13141. doi: 10.1038/s41598-021-92399-2 (PMC8222274; doi:10.1038/s41598-021-92399-2)
Supplement: Supplementary file 1 — Supplementary Information 1. [file 41598_2021_92399_MOESM1_ESM.pdf]

Supplementary Information for: Human Mobility in Response to COVID-19 in France, Italy and UK

Alessandro Galeazzi, Matteo Cinelli, Giovanni Bonaccorsi, Francesco Pierri, Ana Lucia Schmidt, Antonio Scala, Fabio Pammolli, Walter Quattrociocchi

April 19, 2021

Mobility data

For what concerns mobility data, we exploit a dataset built by Facebook with proprietary methods. In particular, the number of people travelling between two locations in a given time interval is provided in two forms: a "baseline" value, that is computed as the average over the 45 days preceding day 0 of data collection for each country, and a "crisis" value that corresponds to near real-time data. Notice that a measurement is retained only if the baseline value exceeds 10, otherwise it is discarded.

Different dismantling strategies

We addressed the impact of different dismantling strategies on node persistence. We consider strategies based on geographical distance (Fig 1) and edge betweenness (Fig 2). It can be observed that both strategies perform significantly worse in approximating the connectedness of the networks. In particular, edge betweenness appears to dismantle the network in very few steps, showing a steep transition to the state where the network is completely disconnected. This is because in the network a lot of edges have betweenness equals to 0 and thus are all disconnected at the same time.

Correlation of economic and demographic indicators with nodes persistence

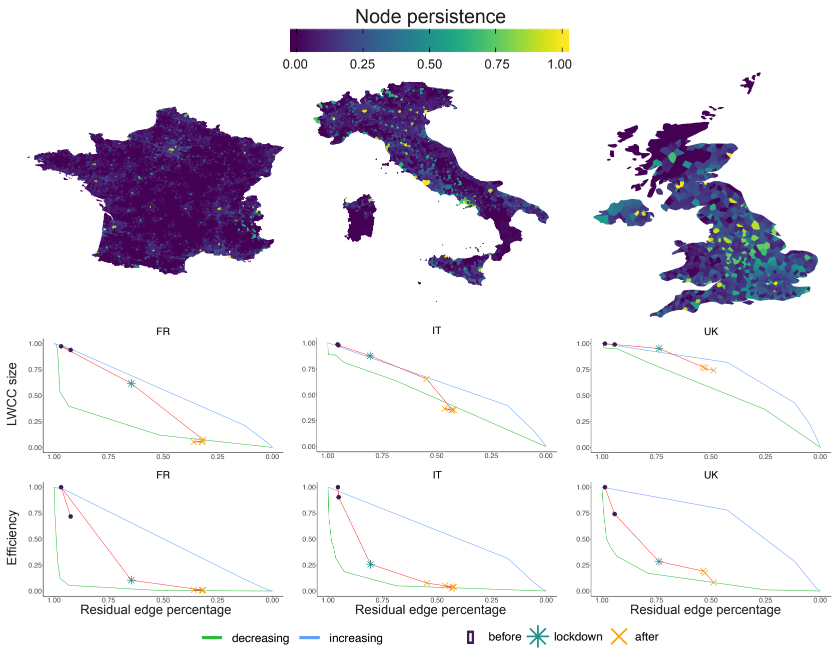

Figure 1: Node persistence and dismantling process based on geographical distance

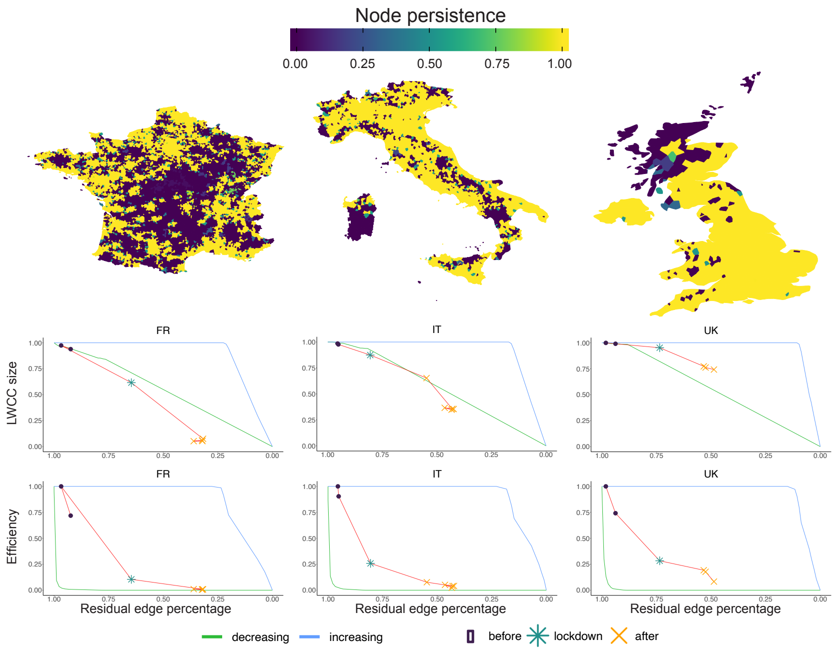

Figure 2: Node persistence and dismantling process based on edge betweenness

	country	All observations Pearson	Spearman	Kendall	obs	Without top 1% Pearson	Spearman	Kendall	obs
Gdp Per Capita (2016)	all	0.3046	0.4154	0.2894	328	0.3732	0.3962	0.2745	324
	ITA	0.4305	0.5374	0.3866	110	0.4305	0.5374	0.3866	110
	FR	0.7751	0.5409	0.3836	93	0.5655	0.5101	0.3574	91
	UK	0.2294	0.2148	0.1529	125	0.1795	0.1964	0.1406	123
		0.0101	*0.0162*	*0.0122*		*0.0469*	*0.0295*	*0.0224*	
Total Population (2019)	all	0.2766	0.3344	0.2273	329	0.2234	0.3179	0.2152	325
	ITA	0.6543	0.4939	0.3581	110	0.3706	0.4507	0.3235	107
	FR	0.6035	0.7921	0.605	94	0.6358	0.7862	0.6001	93
	UK	0.4544	0.5095	0.3603	125	0.4544	0.5095	0.3603	125
Value Added Per Capita (2016)	all	0.398	0.4215	0.2917	328	0.3059	0.402	0.2762	324
	ITA	0.639	0.5987	0.4369	110	0.5433	0.5764	0.4171	108
	FR	0.794	0.7801	0.5954	93	0.6012	0.7655	0.5783	91
	UK	0.4214	0.5203	0.3744	125	0.4214	0.5203	0.3744	125
Population Density (2016)	all	0.5966	0.813	0.622	329	0.6594	0.806	0.6128	325
	ITA	0.8303	0.6612	0.4863	110	0.8303	0.6612	0.4863	110
	FR	0.8292	0.8542	0.6794	94	0.9265	0.8394	0.6588	91
	UK	0.5311	0.6985	0.5079	125	0.517	0.6911	0.4998	124

Table 1: Correlation among node persistence and economic and demographic indicators calculated for all countries together and separately on each country subset. Calculation performed on the full sample (columns on the left) and on the sample without the top percentile to avoid the influence of outliers. First row reports correlation coefficients, second row reports pvalues only for values greater than 0.001.
